# Giant renal angiomyolipomas in a patient with tuberous sclerosis

**DOI:** 10.1590/0100-3984.2016.0082

**Published:** 2018

**Authors:** Carla Lorena Vasques Mendes de Miranda, Camila Soares Moreira de Sousa, Breno Braga Bastos, Carla Valeria Vasques Mendes de Miranda, Francisco Edward Mont'Alverne Filho

**Affiliations:** 1 Med Imagem, Teresina, PI, Brazil; 2 UDI 24 horas, Teresina, PI, Brazil

**Figure 1:**
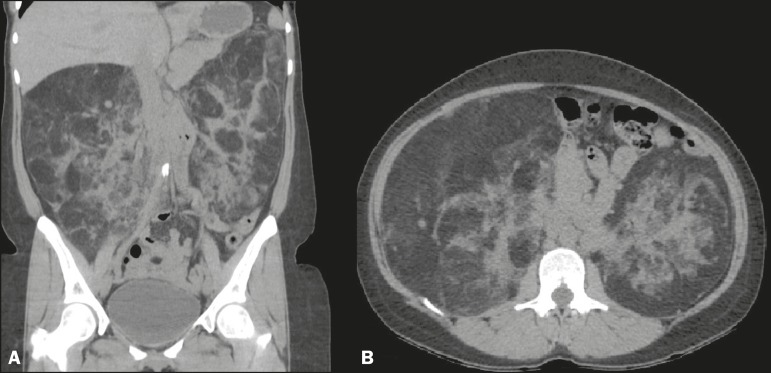
Non-contrast-enhanced CT of the abdomen with coronal reconstruction (**A**) and an axial section (**B**) showing heterogeneous, partially delimited bulky formations with extensive areas of fat attenuation, resulting in marked bilateral occupation of the kidneys, together with diffuse architectural distortion of the parenchyma and local mass effect.

**Figure 2:**
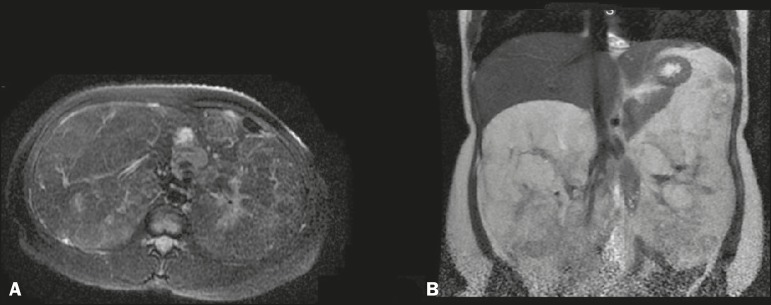
MRI scan of the abdomen showing large, bilateral, heterogeneous, expansile renal formations, with significant signal loss, characterizing fat content, in an axial T2- weighted fat-saturated sequence (**A**). In a coronal T2-weighted fat-saturated sequence there is no significant signal loss (**B**).

Dear Editor,

A 40-year-old female patient, diagnosed 15 years prior with tuberous sclerosis (TS) but not having had periodic follow-up evaluations, presented with a 20-day history of constant abdominal pain. A palpable abdominal mass had been detected during a medical consultation. Laboratory tests revealed no abnormalities. Computed tomography (CT) of the abdomen showed heterogeneous, partially delimited bulky formations with extensive areas of fat attenuation, resulting in marked bilateral occupation of the kidneys, accompanied by diffuse architectural distortion of the parenchyma and local mass effect (Figure 1). Given the clinical history of the patient, the main diagnostic hypothesis was giant renal angiomyolipomas (AMLs), and the correlation with magnetic resonance imaging (MRI) of the abdomen was therefore recommended (Figure 2). Because of the symptom profile associated with extensive bilateral occupation of the kidneys and the high risk of hemorrhage, it was decided that total nephrectomy was the most appropriate therapeutic option for the patient. Postoperatively, the patient was stable and was referred for routine hemodialysis.

Masses of the urinary tract have been the object of a number of recent publications in the radiology literature of Brazil^1-3^. AMLs are rare benign lesions, accounting for 1-3% of all renal tumors, hamartomas being included in the differential diagnosis because of the presence of adipose tissue, neovascularization, and muscle fibers ^4^. Although the most common type of AML is the sporadic form, 10% of cases are associated with TS, with bilateral distribution and, in some cases, multiple masses. In 60% of cases, patients are asymptomatic, the appearance of symptoms and complications being closely related to the size of the tumor; in symptomatic patients, the most common manifestations are abdominal pain and a palpable mass^5-7^.

The diagnosis of AML is typically based on a finding of macroscopic fat in a renal lesion. Classically, AMLs are hyperechoic on ultrasound and are characterized by areas of attenuation below −10 HU on CT. On T1-weighted MRI sequences the areas of fat within the lesions generate signals that are isointense in relation to those of fat present in other organs and hyperintense in relation to those of the renal parenchyma. However, the most reliable diagnosis is based on sequences obtained with and without fat suppression. It is not necessary to include the routine use of intravenous contrast administration in diagnostic and screening protocols for AML^8^. Renal biopsy is not indicated, because it increases the risk of serious complications, as well as because the results rarely alter the approach^4^.

The aim of treatment is the preservation of renal function. Therefore, the options include arterial embolization, radiofrequency ablation, and surgical procedures aimed at maximum preservation of the renal parenchyma, such as enucleation^9,10^.

Surgical intervention becomes necessary when any of the following are identified^5,7^: lesions > 4 cm in diameter; pain; active hemorrhage; changes in the tumor; multiple masses, bilateral masses, or a unilateral mass (in a single kidney); and TS. Total nephrectomy should be reserved for restricted cases^7^ : those in which the majority of the kidney has been occupied by the tumor; those in which a voluminous or solitary lesion is located near the hilum, thus increasing the risk of complications; those in which the results of the imaging examination are inconclusive; those in which there is suspected malignancy; and those in which there is significant retroperitoneal hemorrhage.

In multiple, bilateral renal AMLs accompanied by TS, determining the optimal therapy continues to be a challenge. It is necessary to evaluate the risks for each patient and to establish practices that preserve the renal parenchyma as much as possible. However, in certain cases, such as the one reported here, bilateral nephrectomy is unavoidable.
